# Socio-Emotional Variables Linked to the Consumption of Drugs amongst University Students of Social Sciences: A Pilot Study

**DOI:** 10.3390/ijerph18094502

**Published:** 2021-04-23

**Authors:** José Luis Rodríguez-Sáez, Luis J. Martín-Antón, Alfonso Salgado-Ruiz, Miguel Ángel Carbonero

**Affiliations:** 1 Excellence Research Group GR179 Educational Psychology, Department of Psychology, University of Valladolid, 47011 Valladolid, Spain; luisjorge.martin@uva.es (L.J.M.-A.); miguelangel.carbonero@uva.es (M.Á.C.); 2 Faculty of Psychology, Pontifical University of Salamanca, 37002 Salamanca, Spain; asalgadoru@upsa.es

**Keywords:** emerging adulthood, social support, academic performance, emotional intelligence, consumption of drugs

## Abstract

This descriptive and transversal study, carried out on an intentional sample of 211 subjects who were split in terms of their consumption of psychoactive substances over the last month and who were aged between 18 and 28 (M = 21.36, and SD = 1.90), aimed to explore the emotional intelligence, perceived socio-family support and academic performance of university students vis-à-vis their consumption of drugs and to examine the link between them. The goal was to define university student consumer profile through a regression model using the multidimensional Perceived Social Support Scale (EMAS) and the Trait Meta Mood Scale-24 (TMMS-24) as instruments, together with academic performance and gender. The results report alcohol, tobacco, and cannabis consumption rates that are above the levels indicated by the Spanish household survey on alcohol and drugs in Spain (EDADES 2019) for the 15–34-year-old age range in Castilla y León. A certain link was observed between the consumption of substances and academic performance, although no differences were seen in academic performance in terms of consumer type. There was also no clear link observed between emotional intelligence and academic performance or between social support and academic performance. The predictive contribution of the variables included in the regression model was low (9%), which would advocate completing the model with other predictive variables until more appropriate predictability conditions can be found.

## 1. Introduction

The major cultural, political, and economic changes that have triggered a significant shift in the life cycle of human development, coupled with the economic crisis that has affected developed countries since 2008, have further deepened the negative effects of reaching adulthood. One effect of these changes has been the ever-increasing difficulties youngsters face in fulfilling their expectations of success: leaving the family home at a later age, staying in education longer, and becoming a parent at a later age. In sum, being young today is not the same for young adults as it was for their parents. As a result, some researchers consider that a new stage has emerged in the development of youngsters who live in industrialised societies and that there is a moratorium after secondary education in which individuals, who are no longer teenagers, commence the transition [1,2,3,4,5,6].

This new stage has been conceptualised as emerging adulthood, a stage that occurs between the ages of 18 and 29. Emerging adulthood is defined by five basic characteristics: (1) exploring one’s identity, (2) instability, (3) focusing on oneself, (4) being caught between adolescence and adulthood, and (5) experiencing years of exploration [2]. The period of emerging adulthood enables youngsters to discover and choose the best way to grow when in adult age. This does not exclude the importance of the changes that take place in human development at subsequent stages [7].

In the case of Spain, emerging adults stand out due to the so-called Mediterranean transition period, which is characterised by strong family network involvement in protecting its members from economic and social risks [8,9]. Spanish emerging adults experience greater difficulty in joining the labour market and remain in the family home until after the age of thirty when they go from living in their parents’ home to living with their partner, and then later taking on the role of adults. They also enjoy less independence and can expect greater family support over a longer period, which enables them to try various possibilities in terms of love and work, while enjoying an active social life. In fact, unlike other countries, Spanish emerging adults value their freedom more, in terms of having fewer economic or social responsibilities, than they do their independence [9,10].

### 1.1. Perceived Social Support from the Primary Support Network

Perceived social support is one of the most important protecting factors against the disruptive or adverse experience of starting university [11,12,13]. Although some of the specific situations students face (remaining in their parents’ home, maintaining ties of friendship, etc.) help to achieve a smoother transition, their adaptation will depend to a large degree on how they feel accepted and supported [14].

The parental support that children receive during the transition from adolescence to adulthood is vital and entails a number of benefits [15,16,17]. It is no surprise that the family is seen as the most important source of social support in terms of helping the young to make sense of their lives, followed by their friends [18,19]. It is also not surprising that, when negative, these family ties and interactions are even related to poor academic performance [20] or to emotional distancing linked to enhanced psychological well-being when youngsters perceive a family context that offers little social support [21].

Analysis of the benefits of perceived social support during the transition to higher education has mainly been approached by considering the emotional or academic difficulties that university students might experience [22]. Many empirical studies have found that students who feel they can count on others in times of need display fewer affective problems such as depression, anxiety, or loneliness [23,24,25]. Broadly speaking, the definitions of social support refer to the availability of help through social networks and to how individuals value such a resource [26].

### 1.2. Involvement in Studies

First-cycle university education is no longer valued as highly as it was in the past, and obtaining a university degree has ceased to be seen as providing an advantage when seeking employment or as a guarantee of securing a higher paid job. Such a perception impacts motivation and commitment to studies as well as the desire to continue university studies. Gaining a master’s degree is now seen as a way of standing out (an elitist vision of education) since it is viewed as the time when the skills that are really demanded in the labour market are acquired, beyond the general theoretical education offered by a bachelor’s degree [27]. Furthermore, over half of the students who commence higher education experience difficulties adapting to university life [22,28,29], which is one of the main reasons behind poor academic performance and is why students drop out of university [30,31,32].

Regarding the role played by families in young people’s dedication to their studies, quality family relations that are reciprocal can act as a base that favours greater student involvement and commitment to their studies as well as enhanced academic performance [15,33]. Moreover, in Spain, university students continue in the parental home until they conclude their studies or even longer [9].

Another key factor in achieving academic goals is the role played by peers [15,34,35]. Companionship and social support from peers can be a driving force in youngsters’ motivation and can have a positive impact on their academic performance [35]. Moreover, the young tend to follow the trends set by their circle of friends, which can affect the decisions they take vis-à-vis evidencing a greater or lesser degree of involvement in their studies and with regard to their academic future [34].

In addition to its link to emotional intelligence, academic performance is also associated with the consumption of substances, which can affect the student’s development [36]. Many studies have indeed linked the effects of consuming substances to academic performance [37,38,39].

### 1.3. The Emotional Skills of Young Adults

Regarding the student’s personal development, the notion of emotional intelligence (EI) may be considered in relation to the idea of emotional adjustment, personal well-being, and success in life [36,40,41,42]. The results that emerge from studies confirm how certain shortcomings in emotional intelligence skills can affect various aspects of a person’s life, such as interpersonal relationships, well-being and psychological adjustment [43,44], academic performance [45], and the appearance of disruptive behaviours, including drug consumption [41]. There has even been shown to be inadequate identification, handling, and/or understanding of emotions amongst substance users [41,46,47], both as a result of them paying too much attention to their own emotions and due to difficulty controlling or pinpointing the emotions of others [48]. Recent research has underpinned the idea that a greater understanding of emotional states and a better implementation of strategies that regulate negative emotions are linked to a lower likelihood of engaging in risk behaviour associated with alcohol consumption [49,50]. Nevertheless, further research is required in order to gain a deeper understanding of the links between drug consumption and emotional intelligence [51].

Research has also been carried out into whether emotional intelligence is directly linked to academic performance. The results that emerge concerning such a link remain contradictory. Indeed, early studies conducted with university students reported a direct relation between emotional intelligence and academic performance. Emotional intelligence scores predicted students’ mean academic grades to a significant extent [52]. Subsequently, the results reported by Newsome, Day, and Catano [53] fail to support the positive relations between emotional intelligence and academic performance in Canadian university students. As a result, further inquiry is needed to explore the possible link between these variables due to the contradictory results that have emerged [15,42,54,55].

### 1.4. Drug Use

The most recent data on the use of substances appear in the Spanish household survey on alcohol and drugs in Spain (EDADES, 2019/2020) [56]. Legal substances, alcohol and tobacco, are, in that order, the most commonly consumed drugs. There is also a 15.4% rate of binge drinking over the previous 30 days. The most widely consumed illegal substance is cannabis (10.5% over the last year). The age of first use has shown no significant changes.

The study carried out by Sánchez-Pardo [57] revealed that in Castilla y León, approximately half (48.0%) of youngsters aged between 20 and 29 years old consume alcohol between one and four days a week, whilst a further 11.2% do so daily. Daily average alcohol consumption for youngsters aged between 20 and 29 who are habitual drinkers averages 26.62 g, which is slightly above the 25.25 g recorded for the general population aged between 14 and 70.

Some studies carried out amongst the university population point to a pattern in the consumption of legal drugs that is similar to or slightly higher than the general population [58,59]. Sánchez-Ortega [60] reported that university students consume higher levels of alcohol than the non-university population of the same age and that they also display greater frequency in all kinds of consumption: annual, monthly, and binge drinking. This form of consumption is linked to drinking alcohol “at weekends” or at parties, which is more in line with university students’ preferences. All of this evidences that starting university life entails a stage of experimentation and/or consolidation in consumption [58,61].

As pointed out by García-Mendoza and Parra and Sánchez-Queija [15], this high level of consumption has been linked to a delay in assuming the role of an adult; in other words, with being emerging adults, such that when youngsters take on responsibilities related to work or forming a family, they curb their consumption of substances [62]. Although it may seem that drug consumption is high during the period of emerging adulthood, the family can play a key preventive role, since the presence of a well-functioning family in the years leading up to emerging adulthood is linked to lower levels of substance consumption during emerging adulthood [63,64].

In addition, the social support provided by friends evidences a positive link to indicators of health and well-being and has even been shown to be a protecting factor when coping with the years of change and new situations, such as the transition to emerging adulthood. Excessive levels of consumption in adults are seen to lead to distancing from peer and family groups [15].

### 1.5. The Present Study

The general aim of this work was to explore emotional intelligence, perceived socio-family support, and academic performance of university students who are pursuing a degree in social sciences, in terms of their attitudes and consumption patterns of psychoactive substances, and to determine whether worse results in emotional intelligence are linked to poor performance and greater use of substances and whether perceived low social support is associated with worse academic performance and greater use of substances. The second objective aimed to verify, in accordance with the scientific literature reviewed, whether emotional intelligence, perceived social support, and academic performance prove to be key variables when distinguishing between groups of substance users/non-users.

## 2. Materials and Methods

### 2.1. Sample

We drew on a purposive sample of 211 emerging adults (20 males and 191 females), aged between 18 and 28 (M = 21.36, and SD = 1.90), who were at higher education institutions in Valladolid (UVa) and Salamanca (UPSA). The main descriptive data are shown in Table 1. A total of 28.4% of the university students belonged to the group of non-consumers, 35.1% to the group of consumers, and 36.5% to the group of occasional consumers.

In order to classify the groups, we applied the criterion of inclusion/exclusion depending on reported consumption of substances over the last 30 days:

- Non-consumer group (GNC) (28.4%; N = 60): had not consumed any of these substances over the last 30 days: nicotine (N), cannabis (THC), cocaine (COC), amphetamines (AMP), hypnotics and/or tranquilisers (BZO), hallucinogens (AL), opiates (OPI), inhalants (INH), designer drugs (DD), and other drugs (OD). In addition, they had not drunk alcohol on more than two days (A).
- Consumer group (GC) (35.1%; N = 74): at some time, they had had COC, AMP, BZO, AL, OPI, INH, DD, or OD, or had taken cannabis on more than two days, or had smoked tobacco on 10 or more days, or had drunk alcohol on 10 or more days. There were no restrictions on the amounts of substances consumed.
- Occasional consumer group (GCE) (36.5%; N = 77): made up of students who were not classified in either of the two previous groups. They have therefore been occasional consumers of alcohol (those who had drunk on between three and nine days over the last month), nicotine (had not smoked on more than nine days over the last month), or cannabis (had not taken any on more than two days over the last month).

### 2.2. Evaluation Tools

Socio-demographic variables. This part of the questionnaire comprises different items related to university, degree studies taken, age, sex, etc.

Consumption variables. Consumption of substances was evaluated through a questionnaire designed by Sánchez-Ortega [61] as part of a research project into substance consumption amongst students at the University of Cantabria, based on other questionnaires previously validated and applied to university populations, both in the U.S. and Spain.

Academic variables. Academic performance was evaluated through four items in which participants were asked for the mean grade and final grades obtained at the end of the previous academic year, the number of hours per week they studied, and the number of days they had missed lessons.

Perceived Social Support. The multidimensional Perceived Social Support Scale (EMAS) adapted to Spanish by Landeta and Calvete [65] was used. This is a 12-item instrument that reflects what levels of social support are perceived by those to which the scale is applied. This study used the initial version of Zimet et al. [66], which presents a 7-alternative response scale, where 1 indicates “Totally disagree” and 7 “Totally agree”. Analyses with Spanish populations display suitable internal consistency (relevant persons α = 0.85; family α = 0.89; or friends α = 0.92).

Emotional Intelligence. This was measured through the Trait Meta Mood Scale-24, [67]. This questionnaire is made up of three dimensions: Attention, Clarity, and Mood Repair, with each dimension being measured through eight items that have 5-point Likert-type responses. Analyses of the Spanish population displayed suitable internal consistency (attention α = 0.90; clarity α = 0.90; and mood repair α = 0.86).

### 2.3. Procedure

During the months of October to December 2019, youngsters who were taking a university degree in either Valladolid or Salamanca were contacted and were explained the aims of the study. They were asked to cooperate in the study and, if they agreed to do so, were given an online form with the questions contained in the study. The response rate was 96.3%. There were three people who were discarded because they could not be categorized as emerging adults because of their age; another five people were also not included in the study because they were absent on the day of data collection.

### 2.4. Statistical Analysis 

A comparison was made among the various consumption groups of the following dependent variables: emotional intelligence, perceived social support, and academic performance. Covariance analysis was performed for academic performance, including the days they missed class without justification as the covariables. However, factorial variance analyses were carried out for the various dimensions of emotional intelligence and perceived social support. The Kruskal–Wallis test was applied to gauge the differences between the groups in terms of the risk perception of consuming various substances, including the η^2^_H_ statistic as a size of the effect; considering 0.01 ≤ 0.06 (small effect), 0.06 ≤ 0.14 (moderate effect), and ≥0.14 (large effect). The Mann–Whitney U test was applied as a post hoc analysis to ascertain amongst which groups any differences emerged. All of the comparisons by pairs or multiples were performed post hoc using Bonferroni correction. The relations between emotional intelligence and academic performance, perceived social support and performance, and the indicators for the amount consumed and these variables were analysed using the Pearson bivariate correlation test. Analysis of the links between the indicators of substance consumption frequency and academic performance was performed using the Rho Spearman (rs) bivariate correlation test. For this purpose, we used the statistical package IBM SPSS Statistics, version 26 (2019). All the statistical analyses used showed a 95% confidence level. Finally, in the binary logistic regression models used to determine the profiles of university students who take drugs, the Chi-square test (*p* ≤ 0.05) of the omnibus test was applied to determine the viability of the model. The Cox and Snell R-square test and the Nagelkerke R-square test were used to define the percentage of the dependent variable that predicts the model. Variables were included through forward conditional selection. The Reliability Index (RI) for the Exp (B) was set at 95.5%, and the model’s goodness of fit was tested through the Hosmer–Lemeshow test.

## 3. Results

Results are shown in four sub-sections: consumption of substances, emotional intelligence, academic performance in relation to emotional intelligence, and perceived social support.

### 3.1. Consumption of Substances

The most frequently consumed psychoactive substances were alcohol, tobacco, and cannabis. A total of 97.6% had consumed alcoholic drinks at some point, and 55.5% cannabis. Regarding tobacco, 72% had tried it and 19.9% smoked daily. Amongst the smokers, the mean number of cigarettes/day was 6.23 (SD = 4.5). A total of 22.6% had tried tobacco for the first time when they were between 12 and 13 years of age, and 40.3% when they were between 14 and 15 years of age. A total of 80.1% of the sample had got drunk at some time over the last 12 months, 21% had done so more than 24 times over the last year, and 51.2% had engaged in binge drinking over the last two weeks. A total of 30.6% had tried alcohol for the first time when they were between 12 and 13, and 48.4% when they were between 14 and 15. Regarding the use of the other drugs, consumption was less widespread. Prominent was the use of non-prescription tranquilisers over the last month (1.4%) and other drugs (0.9%). Table 2 shows the principal consumption characteristics of the various substances.

There were no significant differences in consumption patterns in terms of gender. Regarding possible changes in the degree of consumption of illegal substances after starting university, most university students (71.6%) stated that they did not consume, some (13.3%) consumed similar to what they had done previously, 10.4% had increased, and only 4.7% had cut down on their consumption.

The binary logistic regression model that defines the profile of young university students who are drug users (Table 3) is shown below. This gave a Chi-square significance in the omnibus test of *p* = 0.068, such that the model is close to being suitable. Nevertheless, it was only able to explain 9% of the dependent variable, meaning that the model was unacceptable. The Hosmer–Lemeshow test to define the model’s goodness of fit showed *p* = 0.150 (*p* > 0.05), such that it adjusts to reality. The relation between substance use as a dependent variable and the other co-variables obtained statistically significant differences for the relations given with Relevant Person Support and Academic Performance (*p* = 0.019; *p* = 0.009) and with a negative relation being established in both cases (B = −0.468; B = −0.505). The remaining dimensions of Perceived Social Support, Emotional Intelligence, and Sex were not related to substance use.

Finally, Castilla y León university students see as slightly risky behaviour the consumption of marihuana once or twice (40.8%), occasionally (25.1%), or regularly (3.8%); the consumption of cocaine once or twice (12.8%), or regularly (1.4%); trying LSD once or twice (10%); taking amphetamines once or twice (10%) or regularly (0.5%); and drinking alcohol every day, one or two drinks (25.1%), having four or five alcoholic drinks almost every day (5.7%), having five or more alcoholic drinks consecutively (5.2%), or drinking before having sex (16.6%).

When applying the Kruskal–Wallis test (see Table 4), differences were found between the risk perception of different consumption situations depending on whether the student is a consumer (GC), an occasional consumer (GCE), or a non-consumer (GNC), with the size of the effect ranging from small to moderate. Differences in risk perception between the groups (H^2^(2) = 29.923, *p* < 0.001, η^2^_H_ = 0.134) were seen with regard to smoking marihuana occasionally. When applying the Mann–Whitney U test as a post hoc analysis, with the Bonferroni correction, differences were seen to exist between the three groups. The GC displayed the lowest risk perception (Median = 80.4) and the GNC the highest risk perception (Median = 135.7).

### 3.2. Emotional Intelligence 

**Figure 1:**
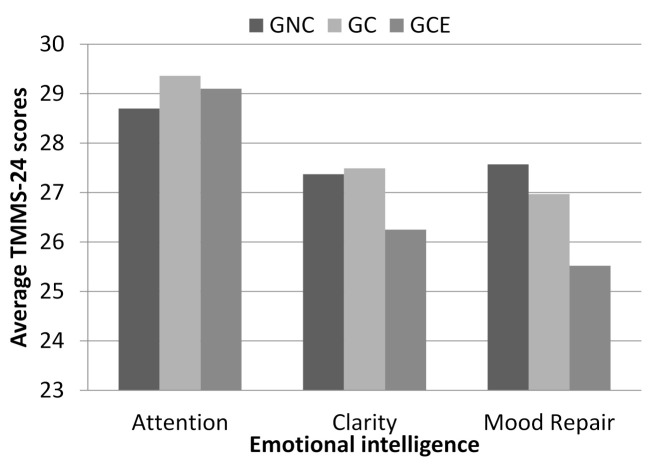
Average of the dimensions of emotional intelligence (TMMS-24) by groups. Note: GNC = Group of non-consumers; GCE = Group of occasional consumers; GC = Group of consumers.

There are no significant differences between the groups in any of the dimensions of emotional intelligence, although worthy of note is the fact that the GNC group displays a higher score in mood repair when compared to the other two groups (see Figure 1).

There are significant negative correlations between the indicators of the amount of various alcoholic drinks consumed and certain dimensions of emotional intelligence: beer on weekdays and clarity (r = −0.146, *p* < 0.05); cognac, anise, *pacharán* (sloe brandy), *orujo* (pomace brandy) on weekdays and clarity (r = −0.136, *p* < 0.05) and mood repair (r = −0.142, *p* < 0.05); *calimocho* (red wine and Coca-Cola) on weekdays and clarity (r = −0.138, *p* < 0.05); whiskey at weekends and mood repair (r = −0.203, *p* < 0.01); and a positive correlation between highball drinks on weekdays and mood repair (r = 0.158, *p* < 0.05). Significant correlations were also observed between the indicators of the consumption frequency of certain substances: tobacco and attention (over the last year, rs = 0.137, *p* < 0.05); cannabis and clarity (over the last month, rs = −0.143, *p* < 0.05); cocaine and attention (over the last year, rs = 0.155, *p* < 0.05).

### 3.3. Academic Performance and Emotional Intelligence

**Figure 2:**
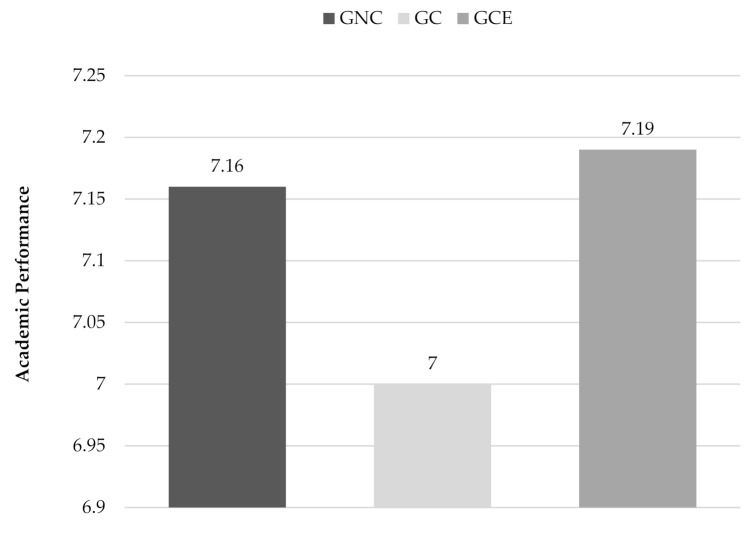
Average grade of the previous academic year in each group related to consumption.

The ANCOVA showed there is a general effect of the absenteeism variable on academic performance, (F(1.205) = 22.917, *p* < 0.001, η^2^_p_ = 0.101), although no differences were found between the groups when taking into account the influence of absenteeism. Figure 2 shows the average grade of the previous academic year for each group.

There is a negative correlation between missing class without justification and the mean academic grade (r = −0.402, *p* < 0.01) and between missing class without justification and the number of hours spent studying each week (r = −0.176, *p* < 0.01).

Differences were found in the proportion of students who pass or fail in relation to the consumption of tobacco (X^2^(2) = 15.259, *p* < 0.001): amongst students who pass, 72.7% do not smoke and 19.2% do so every day. There is also a negative correlation between the average number of cigarettes smoked in one day and academic grades (r = −0.140, *p*< 0.05). There is also a negative correlation between consumption of alcohol and academic performance: beer on weekdays (r = −0.148, *p* < 0.05); beer at weekends (r = −0.137, *p* < 0.05); *calimocho* on weekdays (r = −0.149, *p* < 0.05). Negative correlations were also observed between the indicators of consumption frequency of certain substances and academic performance: tobacco (over the last year, ρ = −0.178, *p* < 0.01; over the last month, ρ = −0.183, *p* < 0.01); cannabis (over the last month, ρ = −0.173, *p* < 0.05); other drugs (over the last month, ρ = −0.160, *p* < 0.05).

A significant correlation was only found between performance and emotional intelligence in the GNC, specifically in the mood repair dimension (r = −0.353, *p* < 0.01) and in the GCE in the attention dimension (r = −0.313, *p* < 0.01).

### 3.4. Perceived Social Support

The descriptive analysis revealed that most participants believed they could count on strong social support (M = 5.99; SD = 1.35). By subscales, scores are very similar (Family M = 5.95; SD = 1.44; Friends M = 5.98; SD = 1.56; Relevant Person M = 6.04; SD = 1.44). There are no differences by type of university, place of residence during the academic year, number of courses failed in the last session, or consumption of substances. Differences did, however, emerge between sexes when estimating perceived social support (*p* < 0.03) and in the social support offered by friends (*p* < 0.01), with women obtaining higher scores.

## 4. Discussion

The aim of this study was to explore emotional intelligence, perceived social support, and academic performance in emerging adults in terms of their consumption of substances and to determine the possible existence of emotional maladjustment or scant social support linked to poorer academic performance. The results of this study point to consumption rates of alcohol, tobacco, and cannabis in university students that are above the levels indicated in the EDADES survey (2019) [68] over the last twelve months for the 15–34-year-old age group in Castilla y León: consumption of alcohol is 8.5 points higher; consumption of tobacco is 6.7 points higher; and the consumption of cannabis is 13 points higher. In addition, the frequency of alcohol intoxication amongst university students over the last 12 months (100% males; 78% females) is much higher than was recorded by EDADES (53.9% and 34.5%, respectively), as well as binge drinking over the last two weeks (60% and 50.3% in male and female university students, respectively, compared to 36.9% and 19.2% in the EDADES survey, over the last month). Something similar happens with the consumption of cannabis, the consumption rate of which over the last 30 days is 8.6 points higher than the sample of 15–64-year-olds in Castilla y León as well as amongst other university samples [69]. In this regard, it is important to point out that consumption of this drug reaches its height during emerging adulthood, 20–25-year-olds, after which consumption tends to remit or disappear [15].

Our results indicate a certain link between the consumption of substances and academic performance, as evidenced in other studies [37,61], although no differences in academic performance can be confirmed in terms of consumer type [70]. There is also no clear link between emotional intelligence and academic performance, although it should be pointed out that habitual consumption of psychoactive substances may be a variable to be taken into account since it is university students in the group of consumers who exhibit excessive attention to their own emotions and who obtain poorer grades. This, therefore, points to an indirect relation between these variables, such that the use/abuse of substances might be an indicator of well-being or psychological stability, a variable which moderates this relation [36,40,71,72].

In addition, no clear relation is seen to exist between social support and academic performance, although these results do seem to suggest that family is a relevant dimension since university students who are not consumers are those who point to greater family support and obtain better grades than the group of consumers. Other studies have found no link between family support and the consumption of tobacco and alcohol but have reported such a link with the consumption of cannabis. It has also been found that emerging adults who perceive greater social support from the family are more dedicated to their university studies [15]. In this regard, the data that emerged from this study confirmed this same relation and pointed to family support as a modulating variable of the consumption of substances on academic performance.

With regard to the hypothetical model of the relation between the socioemotional and substance consumption variables, the predictive contribution of the variables included is low (9%), which would advocate supplementing the model with other predictive variables until more appropriate conditions of predictability are found. Despite not having found empirical support for the hypothesis of whether independent variables (emotional intelligence, perceived social support) can be good predictors of the dependent variable (substance consumption (consumers/non-consumers)), statistically significant differences were found for the relationships given with Relevant Person Support and Academic Performance (*p* = 0.019; *p* = 0.009), establishing a negative relationship in both cases (B = −0.468; B = −0.505). Some authors have found a link between academic performance and substance use [73], and other authors have found a significant relationship (*p* < 0.005) between perceived social support and students’ tendency towards illicit drug use [74]. However, possessing a source of support that enhances personal autonomy can promote drug use. These results can be interpreted as meaning that greater autonomy in young people can lead to greater exposure to risk factors for drug use, greater freedom to explore limits. It would be necessary to determine what level of autonomy can become a risk factor, which could open up a future line of research.

Before concluding, we point to some of the limitations of the present study as well as challenges for future enquiry. Although the study was conducted at two universities located in different areas, future studies will include other public and private universities in the region. Moreover, future studies should draw on the opinion of family and friends so as to compare this with that of emerging adults. Another limitation of our study is the high percentage of women in the sample. This bias might explain why some of the expected differences were not obtained, since women are known to adapt better in the context of consumption.

## 5. Conclusions

The university students who are emerging adults and who took part in the study displayed good academic performance (average grade = 7.1) and reported high levels of support from family and friends. Differences were found in the estimation of perceived social support and social support offered by friends, with women being seen to obtain higher scores.

Data also showed that young adults are keenly aware of the risk to their health of consuming alcohol, despite which their alcohol consumption is above the regional and national average. This might be explained by their being unaware of or failing to attach importance to or not becoming involved in the preventive measures implemented by the universities in question or by other institutions, where such measures exist. This leads us to question the effectiveness or impact of what is being done at universities with regard to preventive measures aimed at reducing the causes that trigger alcohol consumption.

No differences were found to exist in academic performance in terms of consumer type, although the group of consumers did have lower grades on average. Negative correlations (albeit < −0.30) did emerge between the amount (for tobacco) and frequency (for alcohol and cannabis) of certain substances and academic performance. No clear link was observed between emotional intelligence and academic performance. A significant correlation was only found between performance and emotional intelligence in the GNC, specifically in the mood repair dimension, and in the GCE in the attention dimension. Finally, no clear link was observed between social support and academic performance, although it does seem that family support might be a key dimension since university students who are not consumers report greater family support and obtain better grades than the group of consumers.

The nature of our results does not allow us to establish relations of causality. However, we might venture that sensing support from the family could prevent the consumption of illegal drugs such as hashish. In any case, and as pointed out, our correlational study does not allow any relations of causality to be deduced.

Finally, although this study cannot be generalised to university students in Castilla y León as a whole, it might serve as a base for universities and other institutions to design consumption prevention strategies, enhance academic performance, and optimise the development of young university students.

## Figures and Tables

**Table 1:** Socio-demographic features of university students in Castilla y León.

	University Students					
	Male		Female		Total	
	Mean	SD	Mean	SD	Mean	SD
Age	21.85	1.98	21.31	1.88	21.36	1.90
Average academic grade	6.95	0.82	7.13	0.82	7.11	0.82
	Male		Female		Total	
	N	%	N	%	N	%
UVa ^1^	15	75	154	80.6	169	80.1
UPSA ^2^	5	25	37	19.4	42	19.9
Marital status						
Single	20	100	189	99	209	99.1
Married	0	0	2	1	2	0.9
Place of origin						
Valladolid	10	50	95	49.7	105	49.8
Other provinces in CyL ^3^	7	35	60	31.5	67	31.7
Other regions	3	15	31	16.2	34	16.1
Other countries	0	0	5	2.6	5	2.4
Where they live						
Family home	12	60	109	57.1	121	57.3
Rented flat	8	40	66	34.6	74	35.1
Hall of residence	0	0	16	8.3	16	7.6
Only studying and not seeking employment	12	60	104	54.5	116	55
Studying and seeking employment	3	15	39	20.4	42	19.9
Studying and working	5	25	48	25.1	53	25.1

Note: ^1^ University of Valladolid; ^2^ Pontifical University of Salamanca; ^3^ Castilla y León.

**Table 2:** Consumption of addictive substances. University students in Castilla y León, academic year 2019/2020 (N = 211).

Substances	Type of Consumption					
	At Least Once in Your Life		Occasional		Habitual	
	n	%	n	%	n	%
Tobacco	152	72	44 ^a^	20.8	42 ^b^	19.9
Alcohol	206	97.6	196	92.9	174	82.5
Cannabis	117	55.5	68	32.2	34	16.1
Cocaine	3	1.4	2	0.9	0	0.0
Amphetamines	4	1.9	3	1.4	0	0.0
Sedatives/tranquilisers	5	2.4	5	2.4	3	1.4
Hallucinogens	2	0.9	2	0.9	0	0.0
Opiates	1	0.5	1	0.5	0	0.0
Inhalants	2	0.9	1	0.5	0	0.0
Designer drugs	3	1.4	1	0.5	0	0.0
Other drugs	4	1.9	1	0.5	2	0.9

^a^ Occasional smoker: smokes but not every day. ^b^ Daily smoker: at least one cigarette a day. Usual consumer: consumption over the last month; occasional consumption: consumption over the last year.

**Table 3:** Binary logistic regression model of substance consumption.

Drug Use								
Source							95% C.I. for EXP(B)	
	B	S.E.B	Wald	df	*p*	Exp(B)	Lower	Upper
Friends’ support	0.090	0.177	0.258	1	0.612	1.094	0.773	1.549
Family support	0.235	0.183	1.650	1	0.199	1.265	0.884	1.810
Relevant Person Support	−0.468	0.199	5.521	1	0.019	0.626	0.424	0.925
Attention	0.021	0.027	0.631	1	0.427	1.022	0.969	1.077
Clarity	0.011	0.027	0.183	1	0.669	1.011	0.960	1.066
Repair	−0.003	0.025	0.017	1	0.897	0.997	0.949	1.047
Academic Performance	−0.505	0.192	6.920	1	0.009	0.603	0.414	0.879
Sex (1)	−0.227	0.514	0.195	1	0.659	0.797	0.291	2.181
Constant	3.187	1.635	3.802	1	0.051	24.221		

Note: B: coefficient B; S.E.B.: standard error B; df: degrees of freedom; *p*: level of significance; EXP (B): Exponential B; C.I.: confidence interval.

**Table 4:** Comparison of the risk perception of consuming different substances by groups.

	GNC	GCE	GC	X^2^ (2)	*p*	η^2^_H_
	*Mdn*	*Mdn*	*Mdn*			
Trying marihuana once or twice	125.9 ^1^	108.8 ^2^	86.9 ^2^	15.160	0.001	0.063
Smoking marihuana occasionally	135.7 ^1^	107.4 ^2^	80.4 ^3^	29.923	<0.001	0.134
Smoking marihuana regularly	120.6 ^1^	107.6 ^2^	92.4 ^2^	10.306	0.006	0.040
Trying cocaine once or twice	122.1 ^1^	109.8 ^2^	88.9 ^2^	11.918	0.003	0.048
Trying LSD once or twice	118.6 ^1^	108.9 ^2^	92.7 ^2^	7.684	0.021	0.027
Trying amphetamines once or twice	117.9 ^1^	109.8 ^2^	92.3 ^2^	7.803	0.020	0.028
Having one or two alcoholic drinks almost every day	132.1 ^1^	105 ^2^	85.8 ^2^	20.870	< 0.001	0.091
Having four or five alcoholic drinks almost every day	123.9 ^1^	107.9 ^2^	89.5 ^2^	14.936	0.001	0.062
Having five or more alcoholic drinks consecutively	119.4 ^1^	105.4 ^2^	95.8 ^2^	7.125	0.028	0.025
Drinking alcohol before having sex	128 ^1^	95.8 ^2^	98.8 ^2^	12.084	0.002	0.048

GNC = Group of non-consumers; GCE = Group of occasional consumers; GC = Group of consumers.^1,2,3^ Significant difference between groups (in which 1 > 2 > 3). α < 0.05.

## Data Availability

Data from the present study were used with permission of the participants and commitment to data protection, and therefore they are not publicly available.
